# TELLING THE STORY OF ADULT PROTECTIVE SERVICES: CALIFORNIA’S IDENTIFICATION, SERVICES, AND OUTCOMES MATRIX

**DOI:** 10.1093/geroni/igz038.3106

**Published:** 2019-11-08

**Authors:** Pi-Ju ( Liu, Zachary Hass, Kendon Conrad, Karen Conrad, Jarmin C Yeh, Madelyn Iris, Sara Stratton, Andrew Butler

**Affiliations:** 1 Purdue University, West Lafayette, Indiana, United States; 2 the School of Public Health at the University of Illinois at Chicago, Chicago, Illinois, United States; 3 University of Illinois at Chicago, Chicago, Illinois, United States; 4 University of California, San Francisco, California, United States; 5 Northwestern University, Evanston, Illinois, United States; 6 San Francisco Adult Protective Services, San Francisco, California, United States; 7 University of California, Berkeley, Berkeley, California, United States

## Abstract

Adult Protective Services (APS) is responsible for investigating reports of abuse, exploitation, and neglect among vulnerable adults. California’s APS program investigates approximately 15% of all abuse, neglect, and exploitation reports in the country. Once abuse or neglect is substantiated, caseworkers design and implement a service plan for clients to reduce future risk; however, APS intervention effectiveness have not been extensively investigated. In partnership with San Francisco and Napa APS, risk and harm of abuse were measured by type using standardized items from the Identification, Services, and Outcomes Matrix, which is derived from the validated Elder Abuse Decision Support Short Form during case investigation (before APS interventions) and at case closure (after APS interventions). Data from 1,472 older adults’ (on average 78 years old; 57% females) served by APS during the six-month pilot demonstration showed the reduction of risk/harm in self-neglect (p<.001), neglect (p<.001), emotional abuse (p<.001), physical abuse (p=.002), and financial abuse (p<.001) after APS interventions. Effective interventions differ by type of abuse such that caregiver support (b=-.98, p<.10), emergency assistance (b=-1.14, p<.05), and client engagement (b=-1.85, p<.05) decreased self-neglect risk/harm; client engagement (b=-2.24, p<.05) decreased neglect by others risk/harm; case management services (b=-1.17, p<.05) decreased physical abuse risk/harm; and financial planning services (b=-3.99, p<.05) decreased financial abuse risk/harm. No services were identified as effective for emotional abuse. Since effective services differed by type of abuse, it is important to consider the etiology of abuse before implementing the services needed by older adults to effectively decrease harm/risk.

